# Selection and validation of a classification system for a child-centred preference-based measure of oral health-related quality of life specific to dental caries

**DOI:** 10.1186/s41687-020-00268-9

**Published:** 2020-12-09

**Authors:** Helen J. Rogers, Fiona Gilchrist, Zoe Marshman, Helen D. Rodd, Donna Rowen

**Affiliations:** 1 Unit of Oral Health, Dentistry and Society, School of Clinical Dentistry, University of Sheffield, Sheffield, UK; 2 Health Economics and Decision Science, School of Health and Related Research, University of Sheffield, Sheffield, UK

**Keywords:** Caries, Children, Oral health-related quality of life, Utility

## Abstract

**Background:**

Caries Impacts and Experiences Questionnaire for Children (CARIES-QC) is a child-centred caries-specific quality of life measure. This study aimed to select, and validate with children, a classification system for a paediatric condition-specific preference-based measure, based on CARIES-QC.

**Methods:**

First, a provisional classification system for a preference-based measure based on CARIES-QC was identified using Rasch analysis, psychometric testing, involvement of children and parents, and the developer of CARIES-QC. Second, qualitative, semi-structured ‘think aloud’ validation interviews were undertaken with a purposive sample of children with dental caries. The interviewer aimed to identify whether items were considered important and easily understood, whether any were overlapping and if any excluded items should be reintroduced. Interview recordings were transcribed verbatim and thematic analysis conducted.

**Results:**

Rasch analysis identified poor item spread for the items *‘cross’* and *‘school’*. Items relating to eating were correlated and the better performing items were considered for selection. Children expressed some confusion regarding the items *‘school’* and *‘food stuck’*. Parent representatives thought that impacts surrounding toothbrushing (*‘brushing’*) were encompassed by the item *‘hurt’*. Five items were selected from CARIES-QC for inclusion in the provisional classification system; *‘hurt’*, *‘annoy’*, *‘carefully’*, *‘kept awake’* and *‘cried’*. Validation interviews were conducted with 20 children aged 5–16 years old. Participants thought the questionnaire was straightforward and covered a range of impacts. Children thought an item about certain foods being *‘hard to eat’* was more relevant than one about having to eat more carefully because of their teeth and so the *‘carefully’* item was replaced with *‘hard to eat’*.

**Conclusion:**

Following child-centred modification, the preliminary five-item classification system is considered valid and suitable for use in a valuation survey. The innovative child-centred methods used to both identify and validate the classification system can be applied in the development of other preference-based measures.

**Supplementary Information:**

The online version contains supplementary material available at 10.1186/s41687-020-00268-9.

## Background

Dental caries (tooth decay) is a prevalent oral disease, causing significant negative impacts on the lives of children and young people [1, 2]. Whilst pain is the most common feature of caries, there is a growing body of evidence on the further impacts relating to pain on children’s daily lives [3]. These include time off school, difficulty sleeping, speaking, eating and interference with everyday activities [4, 5]. Furthermore, a number of studies have highlighted links between dental caries and general health, with higher levels of untreated dental caries reported to be associated with reduced weight and poor growth [6, 7].

The wider impacts of caries on society are also substantial. In England, approximately 41,558 children aged up to 16-years were admitted to hospital in 2018–2019 with a diagnosis of dental caries [8]. As a result, dental caries remains the most common reason for children to require hospital admission with an estimated annual cost of £39 million to the National Health Service (NHS) [9].

Dental caries is a largely preventable disease and a range of community-based programmes and clinical strategies have been adopted to reduce the prevalence in children. However, there have been few high quality economic evaluations to determine the cost effectiveness of such programmes [10–12]. This creates difficulties for decision-makers and commissioners in determining which interventions to provide within the remit of the NHS [13, 14].

Within child oral health research, this paucity of economic evaluations could be attributed to the lack of a suitable instrument to measure Quality Adjusted Life Years (QALYs) [15]. Presently, only one generic preference-based measure has been used in child oral health research, with limited success; the Child Health Utility 9D (CHU9D) was not found to be sensitive enough to changes in caries status [15, 16]. The lack of use of other measures and the poor psychometric performance of CHU9D suggests that the content of child and adolescent generic preference-based measures may not be appropriate or sensitive for use in oral health research.

There is a clear need for the development of a validated preference-based measure, specifically for children, that is appropriate for measuring treatment benefits for dental caries [15]. This is achievable through the adaptation of a novel child-centred caries-specific oral health-related quality of life (OHRQoL) measure, known as CARIES-QC (Caries Impacts and Experiences Questionnaire for Children) [17]. This measure was developed with involvement of children at every stage, addressing the primary limitation of a number of other measures of OHRQoL [18]. In its current form, CARIES-QC cannot be used to generate QALYs since it is not preference-based. A preference-based measure consists of: a) a classification system that is used to describe the health of all children; and b) a value set used to score all health states described by the classification system.

It is not feasible or practical to gain preference weights from children for all of the twelve items within CARIES-QC [19]. As such, it was necessary to identify a smaller number of items within the measure to form the classification system. Furthermore, the preliminary classification system would require validation with children prior to use in a valuation survey.

This study aimed to identify a classification system for a child-centred preference-based measure using a combination of statistical methodologies and involvement of stakeholders including children, young people and parents. Furthermore, this study sought to validate, and refine where necessary, the preliminary classification system with a sample of children who had experience of dental caries using a qualitative approach. The valuation of the classification system to generate a preference-based measure will be reported elsewhere.

## Methods

Ethical approval for this study was obtained from Yorkshire and the Humber Research Ethics Committee (Reference: 18/YH/0148).

### Identification of the classification system

Several condition-specific preference-based measures have been developed using a staged approach that selects the classification system using a combination of Rasch Analysis, classical psychometric analysis and developer input [20–23]. The present study builds upon this approach by also incorporating child and parent views. The following stages were used to identify the most appropriate items for a classification system:

1. Rasch Analysis
2. Classical psychometric analysis
3. Patient and Public Involvement (PPI)
4. Developer input

The study team discussed the findings of each approach, particularly where stakeholder views were found to conflict with the results of statistical analyses. Where this occurred, agreement was sought by consensus on which items should be selected for inclusion in the preliminary classification system. The final part of this study involved the validation of the preliminary classification system using a qualitative approach. Revisions to the classification system were undertaken accordingly.

### CARIES-QC

The CARIES-QC is a 12-item measure (Table 1) that seeks children’s assessment of the severity of their caries-related impacts, and has been deemed appropriate for use with 5–16 year-olds. The response format of this measure differs from other measures of OHRQoL in that the three levels (‘not at all’, ‘a bit’ and ‘a lot’) relate to the severity, rather than the frequency, of impacts [18]. It has a simple summative scoring system, whereby the difference between each level is assumed to be equal for each item; a response of ‘a bit’ would be assigned one point, ‘a lot’ would score two points, whilst ‘not at all’ suggests the impact has not been experienced and hence a no points are assigned. This instrument has been reported to have “acceptable validity, reliability and responsiveness” [17, 24]. Furthermore, the involvement of children at every stage during the development of CARIES-QC addresses an acknowledged need to view children as active participants within research [25].

**Table 1:** The questions within CARIES-QC (excluding the global question), the related items and severity levels

Questions from CARIES-QC	Items	Levels
How much do your teeth hurt you?	*Hurt*	Not at all, a bit, a lot
Do your teeth make it hard to eat some foods?	*Hard to eat*	Not at all, a bit, a lot
Do you have to eat on one side of your mouth because of your teeth?	Eating on *one side*	Not at all, a bit, a lot
Do you get food stuck in your teeth?	*Food stuck*	Not at all, a bit, a lot
How much do you get kept awake by your teeth?	*Kept awake*	Not at all, a bit, a lot
How much do your teeth annoy you?	Feeling *annoy*ed	Not at all, a bit, a lot
How much do your teeth hurt when you brush them?	Hurt when *brushing*	Not at all, a bit, a lot
Do you have to eat more carefully because of your teeth?	Eat more *carefully*	Not at all, a bit, a lot
Do you have to eat more slowly because of your teeth?	Eat more *slowly*	Not at all, a bit, a lot
Do you feel cross because of your teeth?	Feeling *cross*	Not at all, a bit, a lot
How much have you cried because of your teeth?	*Cried*	Not at all, a bit, a lot
Do your teeth make it hard to do your schoolwork?	Difficulty doing *school*work	Not at all, a bit, a lot

### Data set

The data set for this study came from the original validation study for the CARIES-QC measure, which has been published elsewhere [17]. The data were from a sample of 200 children aged 5 to 16 years who had a diagnosis of active dental caries. Children were asked to complete the CARIES-QC measure at three different timepoints: baseline (T0), prior to the start of treatment (T1) and following a course of dental treatment to manage the caries (T2). Whilst all timepoints were used in the original validation of CARIES-QC, the present study used data from timepoint T0 on which to conduct psychometric and Rasch analyses, as this had the highest number of observations with no attrition. A range of clinical data were also collected to establish the number of teeth affected by caries, whether children reported symptoms, and the pattern of caries (i.e. whether it affected the front teeth) [17].

#### Rasch analysis

Rasch Analysis has been used to convert each participant response onto a latent continuous scale representing the severity of impacts relating to OHRQoL and assesses the spread of responses across the three response levels for each item [20]. Items with a greater spread indicate that the responder is able to distinguish between the item levels and would be stronger candidates for inclusion in the classification system.

In this study, the Rasch Analysis focussed on the spread of items across the three levels (response categories) at logit 0, whereby a greater spread indicated the respondent was able to distinguish between the item levels. Item (χ^2^) goodness-of-fit statistics were also conducted, with the items having the best fit to the underlying model being the best candidates for inclusion in the classification system. Item fit residual scores were also applied in the same way, with those closest to 0 indicating a better fit to the model.

Differential Item Functioning (DIF) was also assessed to determine whether each item was working the same across respondents of different ages, genders, ethnicities and levels of deprivation according to Index of Multiple Deprivation (IMD) scores [26]. Threshold analyses and assessment of local dependencies were also conducted.

Rasch Analysis was conducted using RUMM2030™ software Version 5.3 (©Rumm Laboratory Pty Ltd., Perth, Australia).

#### Classical psychometric testing

Classical psychometric analyses were carried out using SPSS® software (IBM Corporation., New York, United States, Version 24) [20]. Exploratory Factor Analysis was carried out to establish the dimensional structure of CARIES-QC. This was followed by four classical analyses in line with other studies of this type [20, 27].

Firstly, analyses to determine the rate of missing data were undertaken to evaluate item feasibility. Items with more than 5% missing data were considered to be poor candidates for inclusion within the classification system [28].

Internal consistency would usually be determined by comparison of the item with its respective domain score, though in the absence of established domains, correlations between each item with the global question and total score were determined using Spearman’s correlation coefficient. Furthermore, correlations between items were assessed to identify items that were capturing the same aspect of quality of life, where one of the items may be selected in the classification system to reflect the wider set of items.

The distribution of responses was also analysed. Floor and ceiling effects were deemed to be present if more than 15% of participants chose the best (‘not at all’) or worst (‘a lot’) responses [29]. It was acknowledged, however, that in a measure with only three response options, most of the items would have some degree of a floor or ceiling effect, or both. Items with strong floor effects were considered to be poor candidates for the classification system given that they would not be able to capture a deterioration in health. Conversely, items with strong ceiling effects were considered for selection as this suggested an ability to capture the impacts of higher disease severity.

The responsiveness of each item was estimated using the Standardised Response Mean (SRM) in line with similar studies [20, 30]. This was determined to be the most appropriate indicator of effect size given the presence of a correlation greater than 0.5 (Pearson correlation coefficient = 0.529) between baseline (T0) and follow-up (T2) scores [31]. The SRM (also known as Cohen’s d) was calculated by dividing the mean score change (follow-up score (T2) minus the baseline score (T0)) by the standard deviation of the change [31]. The SRMs were interpreted using Cohen’s criteria, whereby < 0.2 is deemed inconsequential, 0.2–0.5 is considered small, 0.5–0.8 is considered moderate and above 0.8 is considered large [32, 33]. A higher SRM indicated greater sensitivity to change.

#### Views of patient and public involvement (PPI) representatives

A panel of children and young people including personal contacts, local schoolchildren and patients from a paediatric dental clinic were invited to give their views at one of two informal meetings held in May and July 2017, to determine their views on the items within CARIES-QC. The panel was comprised of children from a range of ages, genders and ethnicities, with differing experiences of dental caries. This panel was also involved as a steering group for the overall study. These discussions focussed on how important each item was felt to be, whether any items were considered to overlap, and whether any items were felt to be too similar.

Two parent representatives (one mother, one father) were also involved in these discussions, to provide their thoughts on the items within CARIES-QC from their perspectives. The parents were both personal contacts of members of the research team, though had no clinical background. Each parent had two children, one of whom had experience of dental caries. The parent representatives continued to be involved throughout the duration of the study.

#### CARIES-QC development insights

The fourth stage of this process centred on informal discussions with researchers involved in the development of CARIES-QC. It was important to acknowledge any issues or concerns identified by the research team during the development of this instrument, particularly since children were involved at every stage. Furthermore, it was essential that any difficulties surrounding the use of the instrument in different settings and languages were considered.

The findings from these four steps were discussed by the research team, which involved clinicians, a senior health economist, and the researchers who led the development of CARIES-QC. Each approach was weighted equally (i.e. no single approach provided results that were valued more highly than another). The outcome from this meeting was an agreed preliminary classification system.

### Child-centred validation of the preliminary classification system

Validation of the preliminary classification system was undertaken with children and young people who had a diagnosis of dental caries. Potential participants were identified via referral letters received from general dental practitioners at the Paediatric Dental Clinic at the Charles Clifford Dental Hospital, Sheffield. Patients with known diagnoses of dental caries were approached following their initial examination at the dental hospital. A maximum variation purposive sampling approach was used, to ensure participants of different ages, genders, ethnicities and levels of deprivation. Participants were not eligible for inclusion if they were outside of the 5- to 16-year-old age range within which CARIES-QC was developed for. Furthermore, children and parents who were unable to understand spoken and written English language were excluded. A similar approach was used in both formulating the descriptive system and testing the content validity of CARIES-QC [24]. Based on this previous research, it was expected that approximately 20 interviews would be required to reach data saturation.

Parents and children were invited to consent and assent to participate respectively. Qualitative semi-structured interviews were conducted by an experienced qualitative researcher (HJR). A topic guide (see Supplement 1) was used to inform the interviews, which were recorded and transcribed verbatim. Children were asked to ‘think aloud’ whilst completing questions from CARIES-QC within the preliminary classification system whilst the interviewer aimed to determine whether items were considered important, easily understood, and whether any were overlapping [34]. Children were then shown items that were excluded from the preliminary classification system and questioned further to determine whether any should be reintroduced.

Further sociodemographic data, including participant age and ethnicity were also collected. Postcodes were documented to facilitate calculation of the Index of Multiple Deprivation for each participant, given the well-acknowledged relationship between caries experience and socioeconomic deprivation [26, 35]. Clinical caries experience was recorded for each participant, collating the number of decayed, missing and filled primary and permanent teeth, in the form of the dmft and DMFT indices respectively [36].

Simple descriptive statistics were undertaken on the quantitative data. Qualitative data were analysed by two researchers independently (HJR and ZM) using the framework method to inform validation of the classification system, using NVivo 12 (©QSR International Pty Ltd) software for data management. This latter analysis focussed on identifying children’s level of understanding for each item, the amount of importance participants placed upon each item and whether they considered any as redundant or overlapping. PPI representatives for the study were involved in confirming the interpretation of quotes from children and young people were correct. The study team discussed the qualitative findings, which were used to inform modification of the preliminary classification system as required.

## Results

### Identification of the classification system

#### Rasch analysis

The 200 participants from the aforementioned CARIES-QC validation dataset were included in the Rasch analysis, which used the partial credit model. The sociodemographic characteristics and caries experience of the participants in this dataset are provided in Table 2. Overall, the CARIES-QC data were found to have a good item (mean 0.385 ± 0.902) and person fit (mean 0.254 ± 0.999) to the Rasch model.

**Table 2:** Sociodemographic characteristics and caries experience of participants from the original CARIES-QC validation study (dataset used to undertake Rasch analysis and classical psychometric testing in the present study) and the qualitative validation of the preliminary classification system derived from CARIES-QC

	CARIES-QC validation dataset used for Rasch and classical psychometric analyses (*n* = 200)		Qualitative validation of preliminary classification system (*n* = 20)	
**Age (years)**	Mean: 8.1	Range: 5–16	Mean: 10.1	Range: 6–15
**Gender**				
Male	95	(47.5%)	6	(30.0%)
Female	105	(52.5%)	14	(70.0%)
**Ethnicity**				
Asian background	31	(15.5%)	2	(10.0%)
Black background	5	(2.5%)	1	(5.0%)
Mixed background	9	(4.5%)	2	(10.0%)
White British background	130	(65.0%)	14	(70.0%)
Other background	9	(4.5%)	1	(5.0%)
Unknown background	16	(8.0%)	0	(0.0%)
**Socioeconomic status**				
Most deprived	119	(59.5%)	10	(50.0%)
More deprived	37	(18.5%)	0	(0.0%)
Average	20	(10.0%)	3	(15.0%)
Less deprived	13	(6.5%)	3	(15.0%)
Least deprived	11	(5.5%)	4	(20.0%)
**Total dmft**	Mean: 6.24 (SD: 3.45)	Range: 0–16	Mean: 2.85 (SD: 3.05)	Range: 0–12
**Total DMFT**	Mean: 1.57 (SD: 2.18)	Range: 0–13	Mean: 1.7 (SD: 2.88)	Range: 0–11

dmft indicates the number of decayed, missing and filled teeth in the primary dentition, DMFT indicates the number of decayed, missing and filled teeth in the permanent dentition

**Fig. 1:**
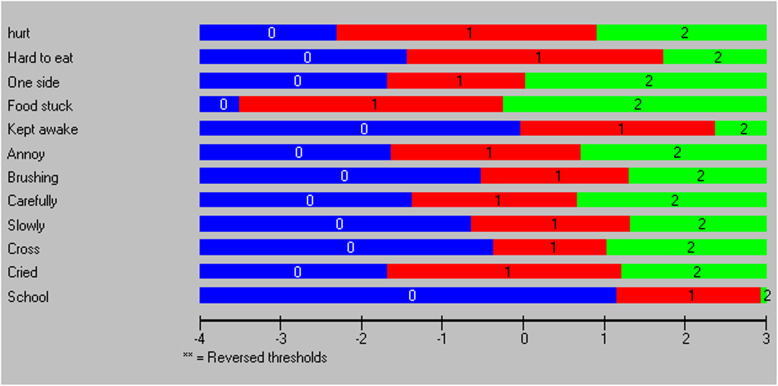
Threshold map for the items within CARIES-QC

Regarding the individual items, none were found to have disordered thresholds (Fig. 1), and none were subjected to local dependency (less than 0.2 above the average correlation) [37].

Table 3 reports the results of the Rasch analysis. The items with the highest spread across the three levels at logit 0 were *‘food stuck’* (1.632), *‘hurt’* (1.605), *‘hard to eat’* (1.585) and *‘cried’* (1.466) respectively. Those with the lowest item spread, and hence candidates for exclusion from the classification system, were *‘cross’* (0.705), *‘one side’* (0.858), *‘school’* (0.894) and *‘brushing’* (0.913) respectively.

**Table 3:** Summary of key results from Rasch Analysis, classical psychometric testing, involvement of PPI representatives and discussions with the developers of CARIES-QC

Item	Item Spread at Logit 0	Item level fit Chi Squared (*P*-value)	Differential Item Functioning (DIF)	Residual	SRM	Disordered thresholds	Missing Data (%)	Floor effects (%)	Ceiling effects (%)	Strong correlations with other items	Concerns from PPI reps	Concerns from CARIES-QC development
**Hurt**	1.605	5.142 (0.076)	✘	−0.757	0.61	✘	✓ (1.5)	✓ (17% ‘a lot’)	✓ (31% ‘not at all’)	✓ (annoy)	✘	✘
**Hard to eat**	1.585	1.288 (0.525)	✓ (age^a^ and gender^b^)	0.048	0.30	✘	✓ (2)	✘	✓ (43% ‘not at all’)	✓ (carefully)	✘	✘
Eating on **one side**	0.858	0.868 (0.648)	✓ (age^b^)	−0.793	0.33	✘	✓ (2.5)	✓ (25% ‘a lot’)	✓ (37% ‘not at all’)	✓ (annoy; carefully)	✓	✘
**Food stuck**	1.632	6.646 (0.036)	✓ (ethnicity^a,^ ^b^)	0.661	0.68	✘	✓ (2)	✓ (32% ‘a lot’)	✘	✘	✓	✓
**Kept awake**	1.202	0.612 (0.736)	✘	−0.393	0.39	✘	✓ (1.5)	✘	✓ (67% ‘not at all’)	✓ (annoy)	✘	✘
Feeling **annoy**ed	1.174	8.699 (0.013)	✘	−1.802	0.93	✘	✓ (2)	✓ (18% ‘a lot’)	✓ (40% ‘not at all’)	✓ (hurt; one side; kept awake; carefully; cross)	✘	✘
Hurt when **brushing**	0.913	1.362 (0.506)	✓ (ethnicity^b^)	0.379	0.49	✘	✓ (0.5)	✘	✓ (57% ‘not at all’)	✘	✓	✘
Eat more **carefully**	1.019	4.367 (0.113)	✘	−1.801	0.38	✘	✘	✓ (18% ‘a lot’)	✓ (43% ‘not at all’)	✓ (hard to eat; one side; annoy; slowly)	✘	✘
Eat more **slowly**	0.988	1.775 (0.412)	✓(deprivation^a^)	−0.874	0.16	✘	✓ 0.5	✘	✓ (55% ‘not at all’)	✓ (carefully)	✓	✘
Feeling **cross**	0.705	2.368 (0.306)	✓ (age^a^ and ethnicity^b^)	0.130	0.51	✘	✘	✘	✓ (59% ‘not at all’)	✓ (annoy)	✓	✘
**Cried**	1.466	4.237 (0.120)	✘	1.112	0.44	✘	✘	✘	✓ (38% ‘not at all’)	✘	✓	✘
Difficulty doing **school**work	0.894	1.339 (0.512)	✓ (ethnicity^a^)	−0.536	0.09	✘	✓ (0.5)	✘	✓ (82% ‘not at all’)	✘	✓	✓

*DIF* differential item functioning, *SRM*:standardised response mean ^a^Uniform DIF ^b^ Non-uniform DIF

Regarding goodness-of-fit, the items ‘*food stuck*’ and *‘annoy’* did not fit the Rasch model at the 5% significance level (*p* = 0.036 and *p* = 0.013 respectively). Conversely, the best-fitting items were *‘hurt’* (χ^2^ = 5.142), *‘carefully’* (χ^2^ = 4.367) and *‘cried’* (χ^2^ = 4.237).

The items ‘*annoy*’ and *‘carefully’* were found to have high negative item fit residuals (− 1.802 and − 1.801 respectively) and the item ‘*cried*’ was found to have a high positive fit residual (1.112). Whilst these are notable, and could potentially indicate item redundancy (associated with Item-Total Correlation), a level of +/− 2.5 should normally be reached for this to cause concern.

The items *‘hard to eat’* (0.031) and *‘cross’* (0.021) were found to have uniform differential item functioning (DIF) with regard to age at the 5% level. *‘Hard to eat’* also showed non-uniform DIF (0.014) at this level, as did *‘one side’* (0.049). The item ‘*food stuck*’ appeared to be working differently for variations in age groups (F = -0.293) and genders (F = -0.126).

#### Classical psychometric testing

Classical psychometric tests were undertaken on the same dataset used for the Rasch analysis (Table 2).

Principal component factor analysis identified only one factor to be present. This factor accounted for 45.54% of the total variance. The high Kaiser-Meyer-Olkin measure of sampling adequacy result of 0.914 determined that the sample was suitable for factor analysis. The statistically significant Bartlett’s Test of Sphericity provided confirmation that the variables were correlated; a degree of correlation is necessary for factor analysis. A Scree plot and further results from the factor analysis can be seen in Supplement 2.

No items were found to have missing values greater than 5% suggesting there were no issues surrounding feasibility [28].

There were moderate levels of correlation (between 0.3 and 0.5) between most items within CARIES-QC (see Supplement 3). Strong correlations (between 0.5 and 0.9) were found between the item *‘annoy’* and five other items, namely *‘hurt’* (r = 0.59), *‘one side’* (r = 0.58), *‘kept awake’* (r = 0.52), *‘carefully’* (r = 0.55), and *‘cross’* (r = 0.51). Similarly the item *‘carefully’* had strong correlations with four other items, namely *‘hard to eat’* (r = 0.51), *‘one side’* (r = 0.63), *‘annoy’* (r = 0.55), and *‘slowly’* (r = 0.60). This suggests that a smaller number of items within the classification system could reflect what is captured by the wider measure. As the factor analysis did not identify multiple domains within CARIES-QC, correlations were undertaken between each item and the global question and total score at baseline (T0). All items had positive correlations with both the global question and the total score.

Regarding the distribution of responses, *‘food stuck’* was the only item to have a floor effect (32% responded ‘a lot’) without also having a ceiling effect. High ceiling effects were noted for *‘kept awake’* and *‘cross’*, with 67% and 59% of respondents reporting no experience of these impacts. A particularly high ceiling effect (82%) was observed in the item *‘school’*, suggesting it was possibly misinterpreted by participants.

Data were available for 38 participants at follow-up (timepoint T2) after receipt of treatment. These data were used to calculate the SRM. The SRM for each item can be seen in Table 3. A strong SRM (> 0.8) was found for *‘annoy’* (0.93), followed by moderate effect sizes for *‘food stuck’* (0.68) and *‘hurt’* (0.61). Trivial effect sizes were observed for *‘school’* (0.09) and *‘slowly’* (0.16).

#### Views of patient and public involvement (PPI) representatives

Children and young people noted that there were multiple items within CARIES-QC relating to eating, and many participants suggested that one item alone could encompass the others on this topic. Children thought the items *‘carefully’* and *‘hard to eat’* had the broadest remit, and that one of these could be considered in place of the rest.

Children expressed some uncertainty about whether the item *‘food stuck’* related to getting food stuck in their teeth in general, or getting food stuck in the holes in their teeth.

Children felt the term *‘annoy’* was too similar to *‘cross’*. Older children in particular thought they would be less likely to use the word *‘cross’*, and hence would prefer the item *‘annoy’*.

Older children thought that their peers would not be likely to admit to crying about their teeth.

Child and parent representatives expressed some confusion about how schoolwork could be affected by teeth. They reasoned that if dental pain was causing the impacts on schoolwork, this may be captured elsewhere under the category of *‘hurt’*.

Parent representatives thought that pain related to toothbrushing, could also come under the umbrella term *‘hurt’*. They also considered whether *‘hurt’* and *‘annoy’* might mean the same thing, though children and young people disagreed.

#### CARIES-QC development insights

The item *‘food stuck’* had translatability concerns when translating into other languages. Anecdotal evidence suggests that children may have a varied understanding of the schoolwork item. These two items could be excluded from the classification on this basis.

Children and young people of different ages viewed the concepts of *‘hurt’* and *‘annoy’* to be different during development of CARIES-QC, although both terms were used to describe the physical sensations that they felt. This suggests it may be important to retain both of these items within the preliminary classification system. In the qualitative research undertaken during the development of the CARIES-QC, older children had admitted to crying about their teeth, in contrast to the suggestion made by the PPI representatives.

##### Discussion of preliminary classification system

The findings from all four steps outlined above were discussed between all members of the study team, and the preliminary classification system was agreed by consensus. A summary of the key discussion points is provided below, based upon the results seen in Table 3.

The items *‘food stuck’* and *‘school’* had issues noted in each of the four steps detailed above, and hence were excluded from the preliminary classification system. As the PPI representatives expressed a need for only one item relating to eating within the classification system, it was felt that eat more *‘carefully’* would encompass this best. This was in part due to its strong correlations with other items regarding impacts and experiences from eating, and its relatively good fit with the Rasch model. Similarly, the item *‘annoy’* was considered important to retain, given its strong correlations with clinical findings. Although parents expressed concerns that *‘annoy’* could be too similar to *‘hurt’*, these items appeared to be independent of each other when analysing the data, and in previous qualitative research children considered them to be separate concepts during the development of the measure [38]. The items *‘cried’* and *‘kept awake’* were considered to be key components of the preliminary classification system, in order to represent the worst states.

Table 4 shows the five items that were selected to form the preliminary classification system, and the broad domains represented by each. The preliminary five-item classification system was then ready for validation with children and young people.

**Table 4:** The preliminary classification system and final validated classification system, with proposed domains

Preliminary classification system	Final validated classification system	Proposed domain
Hurt	Hurt	Physical impacts
Annoy	Annoy	Physical impacts
Carefully	Hard to eat	Impacts on daily activities
Kept awake	Kept awake	Impacts on sleep
Cried	Cried	Emotional impacts

### Validation of the preliminary classification system

‘Think aloud’ interviews were conducted with 20 participants, of which 6 were male, and 14 female, before data saturation was reached. Two potential participants declined to take part; one parent felt their child was too shy to participate, whilst the other reported a lack of time.

The sociodemographic characteristics and caries experience of participants is shown in Table 2. The majority of participants (*n* = 14) were White British, whilst the rest (*n* = 6) were a variety of different ethnicities. The age of participants ranged from 6 to 15 years with a mean of 10 years. Half of the participants (*n* = 10) were found to reside in the most deprived areas of England. All children had active dental caries. The mean dmft was 2.85 (SD 3.05; range 0–12) and DMFT was 1.7 (SD 2.88; range 0–11). The mean length of interview was 8 min and 10 s, though this ranged from below 5 min to upwards of 16 min, with the shortest interviews involving younger children.

The qualitative findings arising from the validation of the classification system are described below, with quotes provided to illustrate each aspect, using participant pseudonyms.

#### Complexity

Children found the questions relating to the preliminary classification system straightforward to complete and did not appear to experience much difficulty in choosing an answer for each question. Furthermore, they believed the questions covered a range of impacts.

*“They’re kind of easy … but they mean a lot”* Jenny, 11 years old.

Children were unsure whether their school friends would be able to answer some of the questions that had been removed from the classification system.

On questioning, younger children struggled to make decisions between items and found it difficult to communicate a clear preference for items capturing similar aspects of health:

“*Both … I like them both*” Lucy, 6 years old.

#### Overlapping items

During the development of the preliminary classification system, parent representatives for the study had raised some concern that the items *‘hurt’* and *‘annoy’* were too similar and potentially overlapping. Nonetheless, these interviews suggest the contrary, as children felt *‘hurt’* and *‘annoy’* described different things, and considered them both to have value.

*“I think they’re very different because annoying and hurt are two different meanings”* Ali, 13 years old.

#### Importance of items

Children had conflicting views on the item *‘cried’* relating to the question ‘have you ever cried because of your teeth?’ Those who had experienced this impact placed greater importance on this item:

*“‘Cause sometimes if they really hurt, I do cry … ..I actually think that is important”* Lucy, 6 years old.

However, those who had never experienced this impact expressed confusion:

*“I don’t really know why people would cry about their teeth”* Lily, 14 years old.

#### Appropriateness of items

Children thought the question ‘do you have to eat more carefully because of your teeth?’ did not adequately describe the dietary restrictions resulting from caries. They displayed a clear preference for one of the questions that had been removed from the classification system, which asked whether their teeth made it hard to eat some foods.

“*If you eat more carefully you can still eat but if you find it hard to eat you can’t really eat much*” Leon, 9 years old.

*“Because if you have to eat more carefully it’s like how you eat whereas “Does your teeth make it hard to eat some foods?” would like eliminate foods out.”* Lily, 14 years old.

### Child-centred modification of preliminary classification system

The findings from the qualitative interviews were then used to inform modifications to the preliminary classification system accordingly.

During the validation interviews, children raised some important issues with the item regarding eating more *‘carefully’*, particularly that it failed to encompass their dietary limitations due to caries. They expressed a clear preference for the item *‘hard to eat’*, and thought this item should be reinserted in the place of the problematic item. The rest of the items within the preliminary classification system were easily understood and considered to be both important and appropriate. Furthermore, children believed the items to be independent of each other, and not overlapping. The final validated classification system can be seen in Table 4.

## Discussion

This paper describes a novel approach to identify a classification system for a paediatric condition-specific preference-based measure from a condition-specific patient-reported outcome measure. The approach taken here builds on the previous approach taken to select items for many condition-specific preference-based measures through the validation of the classification system using qualitative research with children. Furthermore, the methods used to validate the classification system engaged children both as active participants and as experts in their own health.

Children and young people felt that *‘hard to eat’* was a preferable candidate for the classification system compared to *‘carefully’,* as it covered the wider impacts of caries on eating. The decision to replace *‘hard to eat’* for *‘carefully’* within the final classification system was well justified, given that the former had actually outperformed *‘carefully’* in a number of tests conducted in the Rasch analysis. Whilst it lacked the strong correlations with so many other items, its relevance and importance to children and young people was prioritised.

Interestingly, children who had not experienced dental pain severe enough to cause them to cry were unable to understand the relevance of this impact. The range of responses surrounding this item, from a sample who all have diagnosed dental caries, confirms previous research highlighting the variation in impacts that children can experience and how many suffer no symptoms at all [39]. Furthermore, the association between the number of carious teeth and the impacts experienced is often not as linear as one might expect [39]. Nonetheless, it is important for a preference-based measure to contain an item such as *‘cried’*, since this is an impact that is only experienced by those with the greatest severity of the condition. This item, alongside the item *‘kept awake’,* will play an important role in the formation of the worst health state possible within the valuation survey [40, 41].

The systematic and varied approaches used to identify and validate the classification system can be considered one of the strengths of this study. This level of involvement of children and young people is rarely employed in the development of classification systems for paediatric preference-based measures, such as the generic EQ-5D-Y (Euroqol-5 Dimension Youth) and HUI2 (Health Utilities Index 2), or condition-specific measures such as those for atopic dermatitis and asthma [42–45]. Furthermore, whilst qualitative approaches have been used in the identification of items to form classification systems preference-based measures, particularly for older and younger populations, they have not been used in the validation of classification systems [46–48]. This offers many benefits over a quantitative approach, through ensuring that the items within the classification system are considered important to the relevant population. The active involvement of children and young people and the use of a qualitative validation approach could be applied to the future development of paediatric preference-based measures.

Many participants within the validation study lived in areas that were amongst the most deprived in England, which reflects the association between caries prevalence and deprivation [49, 50]. One potential limitation of this study is that it included disproportionately more female participants than males. This does not reflect the wider population, where there is a trend for boys to have a slightly higher prevalence of caries than girls [35]. Similarly, the clinical caries experience (dmft/DMFT) of participants in this study was much higher than the national average of 0.9 [35]. The prevalence of caries in 5-year-old children in Yorkshire and the Humber is known to be greater than the national average (28.5% compared to 24.7% respectively), though this discrepancy is more likely to be explained by the recruitment of participants from a tertiary referral centre. These participants are likely to have been referred to the dental hospital due to the extent of their disease, and resulting symptoms. Whilst this could be considered a limitation of the study due to the lack of representativeness of the sample, it could be argued that those experiencing the impacts described in CARIES-QC would be the most appropriate sample to validate the classification system. Furthermore, this approach ensured that those experiencing the most severe, and perhaps less frequently encountered impacts (e.g. crying) were involved.

This study conducted Rasch analysis and psychometric tests on a dataset with a relatively small sample size, compared to those that have been used in the development of other HRQoL instruments and PBMs, which have seen samples with around 400 to 700 participants being used successfully [20, 51]. Nonetheless, Rasch analysis is known to be sensitive to larger sample sizes, which can cause an increase in the frequency of statistically significant findings, causing difficulties in item reduction [51, 52]. Importantly, the present study was deemed to have a sufficient sample size on which to conduct Factor Analysis.

A range of viewpoints from an interdisciplinary panel were included in the discussions to identify both the preliminary and final classification systems, and hence could be considered a strength of this study. Nonetheless, the reproducibility of this approach is clearly limited, and a different group of researchers may well have selected different items for inclusion in the classification system.

In conclusion, following child-centred modification as detailed above, the preliminary classification system can now be considered valid, since it has been derived taking into account Rasch analyses, classical psychometric tests, PPI and developer input, clinical input, as well as involvement of children with dental caries. The five-item classification system is now suitable for use in a valuation survey with children and young people. This will facilitate generation of QALYs for children with caries, to better inform decision-makers and commissioners regarding the cost-utility of interventions to improve children’s oral health. Furthermore, the innovative methodology used to develop and validate this classification system can be used in the development of other preference-based measures.

## Supplementary Information

**Additional file 1: Supplement 1.** Topic guide used during validation interviews with children and young people

**Additional file 2: Supplement 2 ** Results of Exploratory Factor Analysis.

**Additional file 3: Supplement 3.** Table of correlations between items within CARIES-QC.

## Data Availability

The datasets used and analysed during the current study are available from the corresponding author on reasonable request.

## Abbreviations

CARIES-QC: Caries Impacts and Experiences Questionnaire for Children
CHU9D: Child Health Utility 9 Dimension
DIF: Differential Item Functioning
dmft: Decayed, missing and filled primary teeth
DMFT: Decayed, missing and filled permanent teeth
EQ-5D-Y: Euroqol 5 Dimension - Youth
HUI2: Health Utilities Index 2
IMD: Index of Multiple Deprivation
NHS: National Health Service (UK)
PPI: Patient and Public Involvement
QALY: Quality Adjusted Life Year
SRM: Standardised Response Mean
