# Supplementary material for: Selection and validation of a classification system for a child-centred preference-based measure of oral health-related quality of life specific to dental caries
Source: J Patient Rep Outcomes. 2020 Dec 9;4:105. doi: 10.1186/s41687-020-00268-9 (PMC7726068; doi:10.1186/s41687-020-00268-9)
Supplement: Supplementary file 1 — Additional file 1: Supplement 1. Topic guide used during validation interviews with children and young people [file 41687_2020_268_MOESM1_ESM.docx]

Supplement 1: Topic guide used during validation interviews with children and young people

**Developing a caries-specific child-centred utility measure**

**A quiz about teeth: what do you think?**

**Topic Guide**

**Introduction**

- Complete assent/consent forms for participant and parents/carers
- Complete socio-demographic data collection form
- Purpose is to talk to children and young people about a questionnaire which was made to help us find out what it’s like to have holes in your teeth
- Children/young people will be asked to fill in the questionnaire. They will then be asked to ‘think aloud’ whilst completing it. They will also be asked some more questions about the questionnaire at the end
- Interview will last as long as they wish, but on average 10 minutes
- Use of a digital recorder by researcher, and the talk will later be written up
- Not a test, and no right or wrong answers (child/young person is the expert)
- Can ask for help with reading or filling in the quiz/questionnaire at any time
- Doesn’t have to talk about anything they don’t want to
- Participation is voluntary and children can change their mind and stop the interview at any point. Agree a stop signal (e.g. hand in the air, saying “stop”)
- Answers will be private

**Think Aloud task prompts**

- What are you thinking now?
- What are your thoughts about this page?
- Can you tell me more about that?

**Questionnaire usability**

- What did you think about the questionnaire?
- Go through each question and ask if participant found it easy, OK, or hard to answer and understand
- Can you see any problems with the questionnaire?

**Removal and reintroduction of questions**

- Were there any questions in the quiz that you didn’t like? Why didn’t you like them?
- Were there any questions in the quiz that you did like? Why did you like them?
- Were there any questions that you would want to take out of the quiz?
- Show participant questions that were removed from CARIES-QC
- Do you think any of these questions are important?
- Do you think any of these questions should be put into the questionnaire?

**Next steps**

- Thank the participant
- Reassurance again about confidentiality/privacy
- Explain that findings will ultimately be used to develop a questionnaire to help us find out which dental treatments are value-for-money. We will send all the participants a report so they know what we found out
- Ask the child and parent whether they would prefer the report to be sent to them via post or email
- Give the participant a gift voucher and ask them to sign reciept
