# Supplementary material for: Selection and validation of a classification system for a child-centred preference-based measure of oral health-related quality of life specific to dental caries
Source: J Patient Rep Outcomes. 2020 Dec 9;4:105. doi: 10.1186/s41687-020-00268-9 (PMC7726068; doi:10.1186/s41687-020-00268-9)
Supplement: Supplementary file 2 — Additional file 2: Supplement 2 Results of Exploratory Factor Analysis. [file 41687_2020_268_MOESM2_ESM.docx]

Supplement 2: Results of Exploratory Factor Analysis

Supplementary Table 1: Results from Kaiser-Meyer-Olkin measure of sampling adequacy and Bartlett's Test of Sphericity

| **KMO and Bartlett's Test** |  |  |
| --- | --- | --- |
| **Kaiser-Meyer-Olkin Measure of Sampling Adequacy** |  | 0.914 |
| **Bartlett's Test of Sphericity** | Approx. Chi-Square | 910.797 |
|  | df | 66 |
|  | Sig. | 0 |

Supplementary Table 2: Explanation of total variance

| **Component** | **Initial Eigenvalues** | | **Extraction sums of squared loadings** | |
| --- | --- | --- | --- | --- |
|  | Total % of cumulative variance % | | Total % of cumulative variance % | |
| **1** | 5.585 46.54 | 46.54 | 5.585 | 46.54 46.54 |
| **2** | 0.963 8.025 | 54.566 |  |  |
| **3** | 0.845 7.039 | 61.605 |  |  |
| **4** | 0.75 6.248 | 67.853 |  |  |
| **5** | 0.685 5.708 | 73.561 |  |  |
| **6** | 0.633 5.275 | 78.836 |  |  |
| **7** | 0.576 4.798 | 83.634 |  |  |
| **8** | 0.504 4.202 | 87.836 |  |  |
| **9** | 0.459 3.822 | 91.658 |  |  |
| **10** | 0.397 3.308 | 94.966 |  |  |
| **11** | 0.311 2.594 | 97.56 |  |  |
| **12** | 0.293 2.44 | 100 |  |  |


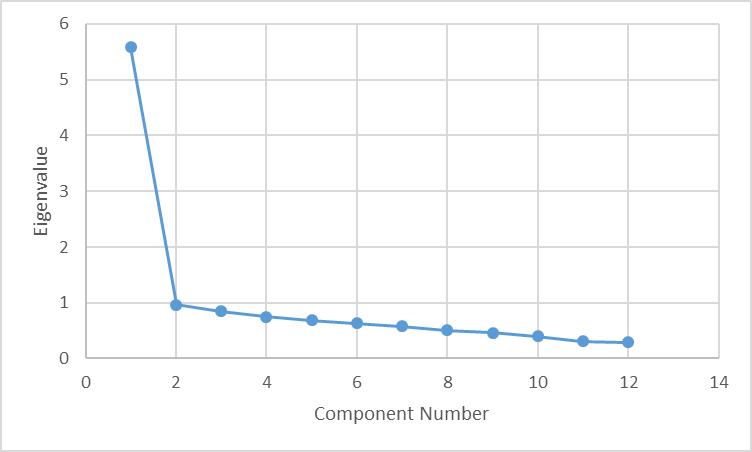


Supplementary Figure 1: Scree plot from Principal Component Analysis, demonstrating the presence of one factor
