# Supplementary material for: Selection and validation of a classification system for a child-centred preference-based measure of oral health-related quality of life specific to dental caries
Source: J Patient Rep Outcomes. 2020 Dec 9;4:105. doi: 10.1186/s41687-020-00268-9 (PMC7726068; doi:10.1186/s41687-020-00268-9)
Supplement: Supplementary file 3 — Additional file 3: Supplement 3. Table of correlations between items within CARIES-QC. [file 41687_2020_268_MOESM3_ESM.docx]

Supplement 3: Table of correlations between items within CARIES-QC

| **Correlations** | | | | | | | | | | | | | | |
| --- | --- | --- | --- | --- | --- | --- | --- | --- | --- | --- | --- | --- | --- | --- |
|  | | | hurt | hard to eat | one side | food stuck | kept awake | annoy | brushing | carefully | slowly | cross | cried | school |
| Spearman's rho | hurt | Correlation Coefficient | 1.000 | .397^**^ | .450^**^ | .386^**^ | .466^**^ | .591^**^ | .407^**^ | .439^**^ | .469^**^ | .390^**^ | .494^**^ | .390^**^ |
|  |  | Sig. (2-tailed) | . | .000 | .000 | .000 | .000 | .000 | .000 | .000 | .000 | .000 | .000 | .000 |
|  |  | N | 197 | 196 | 195 | 196 | 197 | 196 | 196 | 197 | 196 | 197 | 197 | 196 |
|  | hard to eat | Correlation Coefficient | .397^**^ | 1.000 | .491^**^ | .308^**^ | .298^**^ | .375^**^ | .367^**^ | .509^**^ | .497^**^ | .360^**^ | .315^**^ | .346^**^ |
|  |  | Sig. (2-tailed) | .000 | . | .000 | .000 | .000 | .000 | .000 | .000 | .000 | .000 | .000 | .000 |
|  |  | N | 196 | 196 | 194 | 195 | 196 | 195 | 195 | 196 | 195 | 196 | 196 | 195 |
|  | one side | Correlation Coefficient | .450^**^ | .491^**^ | 1.000 | .420^**^ | .363^**^ | .583^**^ | .335^**^ | .625^**^ | .480^**^ | .427^**^ | .366^**^ | .296^**^ |
|  |  | Sig. (2-tailed) | .000 | .000 | . | .000 | .000 | .000 | .000 | .000 | .000 | .000 | .000 | .000 |
|  |  | N | 195 | 194 | 195 | 194 | 195 | 194 | 194 | 195 | 194 | 195 | 195 | 194 |
|  | food stuck | Correlation Coefficient | .386^**^ | .308^**^ | .420^**^ | 1.000 | .260^**^ | .465^**^ | .377^**^ | .397^**^ | .294^**^ | .318^**^ | .329^**^ | .236^**^ |
|  |  | Sig. (2-tailed) | .000 | .000 | .000 | . | .000 | .000 | .000 | .000 | .000 | .000 | .000 | .001 |
|  |  | N | 196 | 195 | 194 | 196 | 196 | 195 | 195 | 196 | 195 | 196 | 196 | 196 |
|  | kept awake | Correlation Coefficient | .466^**^ | .298^**^ | .363^**^ | .260^**^ | 1.000 | .521^**^ | .353^**^ | .413^**^ | .384^**^ | .279^**^ | .402^**^ | .452^**^ |
|  |  | Sig. (2-tailed) | .000 | .000 | .000 | .000 | . | .000 | .000 | .000 | .000 | .000 | .000 | .000 |
|  |  | N | 197 | 196 | 195 | 196 | 197 | 196 | 196 | 197 | 196 | 197 | 197 | 196 |
|  | annoy | Correlation Coefficient | .591^**^ | .375^**^ | .583^**^ | .465^**^ | .521^**^ | 1.000 | .375^**^ | .552^**^ | .451^**^ | .510^**^ | .465^**^ | .392^**^ |
|  |  | Sig. (2-tailed) | .000 | .000 | .000 | .000 | .000 | . | .000 | .000 | .000 | .000 | .000 | .000 |
|  |  | N | 196 | 195 | 194 | 195 | 196 | 196 | 195 | 196 | 195 | 196 | 196 | 195 |
|  | brushing | Correlation Coefficient | .407^**^ | .367^**^ | .335^**^ | .377^**^ | .353^**^ | .375^**^ | 1.000 | .471^**^ | .409^**^ | .323^**^ | .327^**^ | .278^**^ |
|  |  | Sig. (2-tailed) | .000 | .000 | .000 | .000 | .000 | .000 | . | .000 | .000 | .000 | .000 | .000 |
|  |  | N | 196 | 195 | 194 | 195 | 196 | 195 | 199 | 199 | 198 | 199 | 199 | 198 |
|  | carefully | Correlation Coefficient | .439^**^ | .509^**^ | .625^**^ | .397^**^ | .413^**^ | .552^**^ | .471^**^ | 1.000 | .599^**^ | .442^**^ | .414^**^ | .304^**^ |
|  |  | Sig. (2-tailed) | .000 | .000 | .000 | .000 | .000 | .000 | .000 | . | .000 | .000 | .000 | .000 |
|  |  | N | 197 | 196 | 195 | 196 | 197 | 196 | 199 | 200 | 199 | 200 | 200 | 199 |
|  | slowly | Correlation Coefficient | .469^**^ | .497^**^ | .480^**^ | .294^**^ | .384^**^ | .451^**^ | .409^**^ | .599^**^ | 1.000 | .343^**^ | .354^**^ | .377^**^ |
|  |  | Sig. (2-tailed) | .000 | .000 | .000 | .000 | .000 | .000 | .000 | .000 | . | .000 | .000 | .000 |
|  |  | N | 196 | 195 | 194 | 195 | 196 | 195 | 198 | 199 | 199 | 199 | 199 | 198 |
|  | cross | Correlation Coefficient | .390^**^ | .360^**^ | .427^**^ | .318^**^ | .279^**^ | .510^**^ | .323^**^ | .442^**^ | .343^**^ | 1.000 | .354^**^ | .356^**^ |
|  |  | Sig. (2-tailed) | .000 | .000 | .000 | .000 | .000 | .000 | .000 | .000 | .000 | . | .000 | .000 |
|  |  | N | 197 | 196 | 195 | 196 | 197 | 196 | 199 | 200 | 199 | 200 | 200 | 199 |
|  | cried | Correlation Coefficient | .494^**^ | .315^**^ | .366^**^ | .329^**^ | .402^**^ | .465^**^ | .327^**^ | .414^**^ | .354^**^ | .354^**^ | 1.000 | .330^**^ |
|  |  | Sig. (2-tailed) | .000 | .000 | .000 | .000 | .000 | .000 | .000 | .000 | .000 | .000 | . | .000 |
|  |  | N | 197 | 196 | 195 | 196 | 197 | 196 | 199 | 200 | 199 | 200 | 200 | 199 |
|  | school | Correlation Coefficient | .390^**^ | .346^**^ | .296^**^ | .236^**^ | .452^**^ | .392^**^ | .278^**^ | .304^**^ | .377^**^ | .356^**^ | .330^**^ | 1.000 |
|  |  | Sig. (2-tailed) | .000 | .000 | .000 | .001 | .000 | .000 | .000 | .000 | .000 | .000 | .000 | . |
|  |  | N | 196 | 195 | 194 | 196 | 196 | 195 | 198 | 199 | 198 | 199 | 199 | 199 |
| **. Correlation is significant at the 0.01 level (2-tailed). | | | | | | | | | | | | | | |
